# Oral Health and Hygiene Status of Global Transgender Population: A Living Systematic Review and Meta-Analysis

**DOI:** 10.3390/ijerph22030433

**Published:** 2025-03-14

**Authors:** Vaibhav Kumar, Jasleen Thakker, Abhishek Royal, Nikhil Bhanushali, Ziad D. Baghdadi

**Affiliations:** 1 Department of Public Health Dentistry, Dr. G.D. Pol Foundation YMT Dental College and Hospital (Affiliated to Maharashtra University of Health Science, Nashik), Kharghar, Navi Mumbai 410210, India; 2 Kartavya Disha Global Foundation, Vasai, Maharashtra 401202, India; 3 Independent Public Health Consultant, New Delhi, India; 4 Department of Public Health Dentistry, Terna Dental College, Navi Mumbai 400706, India; 5 Department of Preventive Dental Sciences, Division of Pediatric Dentistry, University of Manitoba, Winnipeg, MB R3E 0W2, Canada

**Keywords:** dental hygiene status, malocclusion, oral mucosal lesions, oral health status, periodontal status, transgender

## Abstract

Due to several interpersonal, social, and organizational challenges, dental health has been occasionally compromised in the transgender population. There is a lack of awareness among transgender persons to access affordable trans-competent oral health care. More information is required to identify and assess the oral health condition of this population in order to encourage better access to oral healthcare and effectively influence public health policy and practice. This systematic review aims to provide evidence about the status of oral health and hygiene of the transgender population across the globe. A systematic literature search using keywords and MESH search terms was conducted using PubMed, Medline, Google Scholar, and EBSCO online databases. The references of included journal articles were manually searched and appropriate studies were included, which were then critically appraised using the Joanna Briggs Institute (JBI) tool and the Newcastle–Ottawa protocol for the risk of bias assessment of prevalence studies, with each study assessed by two independent reviewers. Based on the search procedures, a total of 2026 articles were initially screened and, after evaluation, 20 were included in the systematic review. Transgender persons often face stigma and discrimination in dental healthcare settings, which affects their oral health status. A greater prevalence of substance abuse stemming from anxiety, lack of adequate education, and poor socioeconomic status leads to an increased prevalence of oral health diseases in this marginalized population. There is a need for policies and reforms to appraise their oral health and hygiene status and improve access to oral health services in this population.

## 1. Introduction

Bockting, in the year 1999, first defined transgender persons as “individuals who cross or transcend culturally defined categories of gender.” [1]. Globally, there is outstanding dynamism in dismantling stereotypical barriers concerning the transgender population to create a receptive society in which the ability to express one’s gender is normal and not a privilege. For decades now, this community has been struggling to breach the prejudice they are subjected to in the form of violence, harassment, unacceptability, and denial of human rights, and the sphere of health care services and dental health care is no exception [1].

According to the World Health Organization (WHO), oral health is defined as a state of being free of mouth and facial pain, oral infections and sores, and oral and other diseases that limit an individual’s capacity to bite, chew, smile, and speak, as well as their psychosocial well-being. The importance of oral health in governing an individual’s overall health, well-being, and quality of life is indispensable. Oral diseases commonly comprise dental decay, periodontal disease, tooth loss, and oral cancer, including oral manifestations of HIV infection, which are the commonest of chronic diseases and sustain a significant portion of public health problems because of their prevalence, their repercussions on the comprehensive health of individuals and society, and hefty expenses associated with their treatment [2].

Many obstacles in areas as diverse as social, financial, interpersonal, and health eventually affect how transgender people view getting medical care. At a social level, their unacceptance puts them through emotional instability, shame, fear, and internalized transphobia. Poor health literacy, lack of familiarity with the health care system, facilities, and insurance, as well as financial restrictions, contribute to an increased prevalence of HIV and drug abuse, including alcohol and tobacco use. Transgender individuals face discrimination by health care workers, with inadequate consideration, knowledge, and empathy, verbal and physical harassment, and direct refusal or reluctance to provide treatment [3,4]. Dental care professionals are reported to deny oral health services more likely than physicians, considering their sexual orientation and gender expression with greater apathy [5]. In a study conducted by Heima et al. to assess dental fear among transgender individuals, 14.2% of participants indicated they were “very much” or “extremely” fearful of experiencing maltreatment in a dental clinic [6]. Lack of competent training, decrepit curriculum, and a deficiency of receptive care environments among dental professionals eventually contribute to increased risk of poor oral health.

The transgender population faces significant barriers in accessing equitable healthcare, particularly in oral health, a domain that has received little attention in research and policy discourse. Discrimination, financial instability, and lack of gender-affirming care contribute to significant disparities in oral health outcomes. Despite growing advocacy for transgender rights, there is a persistent knowledge gap regarding their oral health status on a global scale.

As the field of transgender health research continues to expand, there is a growing need for dynamic and adaptive evidence synthesis methods. A promising approach is the concept of ‘living systematic reviews’, which involve periodic updates to integrate new findings in real time. Unlike traditional systematic reviews that provide a snapshot of available evidence at a fixed point in time, living systematic reviews continuously evolve, ensuring that healthcare professionals, researchers, and policymakers have access to the most current data. Implementing this approach for transgender oral health research would allow for a more responsive and up-to-date understanding of disparities, risk factors, and intervention strategies, ultimately fostering more inclusive and equitable healthcare policies.

This living systematic review and meta-analysis aims to fill this gap by synthesizing the available global evidence on the oral health and hygiene status of transgender individuals, such as dental caries, periodontitis, oral lesions, tooth loss, and oral hygiene status.

The central hypothesis guiding this review is that transgender individuals experience significantly poorer oral health outcomes than cisgender populations, driven by a combination of social stigma, economic challenges, and barriers to healthcare access. By systematically evaluating the existing literature, this study seeks to identify patterns, quantify disparities, and highlight areas requiring further research and policy intervention.

## 2. Materials and Methods

**Figure 1:**
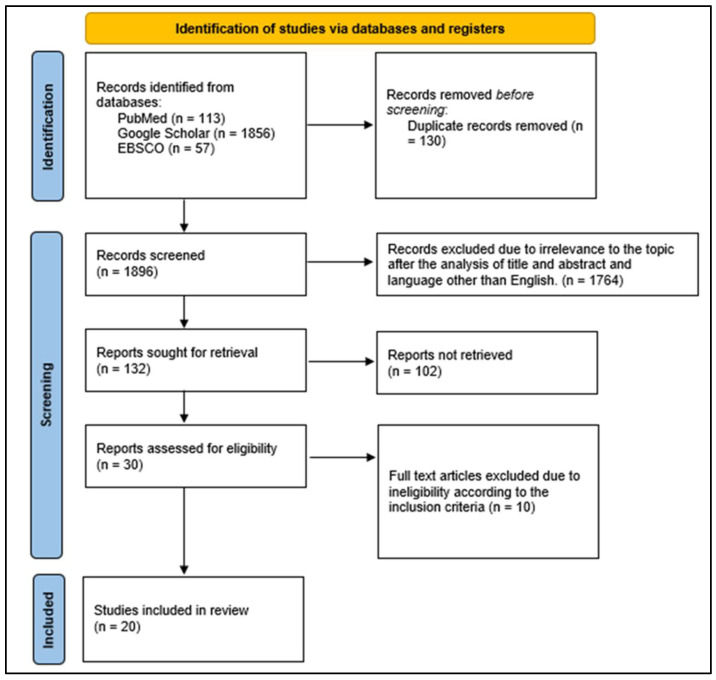
PRISMA flowchart—identification of studies via databases and registers.

This living systematic review and meta-analysis has been structured in agreement with the Preferred Reporting Items for Systematic Reviews and Meta-Analyses (PRISMA)-E 2012 checklist and is registered on PROSPERO (International Prospective Register of Systematic Reviews) (protocol number: CRD42021272384) [7]. An ethics review was not mandated since this was a systematic review that was based on the compilation of secondary literature from the articles included in the study (Figure 1).

It was conducted with the hypothesis that transgender individuals experience disproportionately poorer oral health outcomes due to structural barriers and social determinants of health. Our study design aimed to identify and quantify disparities in oral health outcomes across different transgender subgroups, assess patterns in healthcare access, and highlight policy gaps. The review was structured to address key research questions, including: (1) What are the primary oral health challenges faced by transgender individuals globally? (2) How do these disparities compare to those observed in cisgender populations? (3) What factors contribute to these disparities, and what policy interventions can mitigate them?

*Population* (P): Individuals who identify themselves as transgender.

*Outcome* (O): Evaluating the oral health and dental hygiene status among the transgender population, including their oral hygiene practices; oral hygiene status as measured by the Oral Hygiene Index Simplified (OHI-S); periodontal status as measured by the Calculus Index (CI), Plaque Index (PI), Clinical Attachment Loss (CAL), Community Periodontal Index and Treatment Needs (CPITN), Modified Gingival Index (MGI), Modified Community Periodontal Index (M-CPI), and Probing Pocket Depth (PPD); dental caries as measured by the Decayed-Missing-Filled-Treatment index (DMFT) and any other modes of measurement used; malocclusion; premalignant lesions; and any other relevant oral findings.

*Study design* (S): The studies which have been considered are observational cross-sectional (descriptive studies) and cohort studies and original research papers.

### 2.1. Inclusion and Exclusion Criteria

The studies of interest were all descriptive studies conducted among the transgender population to assess their oral health, hygiene, and diseases. The articles were restricted to those published until August 2021 in English and available in full text only. Table 1 illustrates the summary of the included articles.

The articles without full texts, incomplete information to answer the question of interest, and published in a language other than English where translation into English was not available were excluded. An attempt was made to distinguish any unpublished articles and contact published study authors for additional/missing information. The excluded articles and the reasons for their exclusion are shown in Table 2.

### 2.2. Search Strategy

Free text words and MeSH terms were utilized, comprising the headings of ((Transgender) OR (transsexual) OR (eunuch) OR (lgbtq) OR (Third gender) OR (Sexual minority)) AND ((Dentition status) OR (Dental caries) OR (Tobacco) OR (periodontal status) OR (Clinical attachment loss) OR (Calculus index) OR (Plaque index) OR (Pocket depth) OR (Oral hygiene habits) OR (Oral hygiene status) OR (Oral cancer) OR (oral mucosal lesions) OR (malocclusion) OR (Dental decay)).

### 2.3. Studies with Outdated or Inappropriate Terminology Such as ‘Eunuchs’

Excluding studies solely based on outdated terminology would introduce selection bias, potentially leading to an incomplete representation of transgender oral health status in certain regions. Some older studies and studies from specific cultural contexts used terms such as “eunuchs” or other terminology that, while now considered outdated or inappropriate, historically referred to populations within the broader transgender spectrum.

In many countries, particularly in South Asia (e.g., India, Pakistan, and Bangladesh), legally recognized third-gender identities (e.g., Hijra communities) have been studied under various terminologies in both academic and clinical literature. While these terms may not align with modern transgender identity classifications, they represent a significant population group that experiences oral health disparities.

Completely excluding studies based on terminology rather than methodological rigor would result in a significant loss of valuable data, particularly from regions where contemporary transgender health research is limited.

### 2.4. Mitigation Strategies to Address Terminology Concerns

To ensure inclusivity and accuracy while maintaining methodological rigor, the following steps were undertaken:

1. Standardization of Terminology in Data Synthesis: Regardless of the terminology used in the original studies, all extracted data were systematically categorized under standardized, inclusive terminology in our analysis and discussion.
2. Transparent Reporting and Acknowledgment: We explicitly acknowledge that certain included studies employed outdated terminology and provided necessary clarifications to avoid misrepresentation or insensitivity.
3. Sensitivity Review and Ethical Considerations: The manuscript has undergone a review to ensure that our synthesized findings use affirming and appropriate language in line with contemporary standards in transgender health research.

### 2.5. Search Databases

An electronic literature search of PubMed, Medline, Google Scholar, and EBSCO online databases was conducted from September 2021 to November 2021.

### 2.6. Data Extraction

Two reviewers independently assessed the search results based on the title and abstract for initial screening. They then accessed and screened the full texts of the eligible articles. In case of discrepancies in data extraction, both reviewers developed consensus before making a decision. Rayyan QCRI software was used to remove duplicates, and MS Excel 2010 was used to store the data.

The following details were extracted from the included articles: author, year of publication, population characteristics, assessment tool used, outcome measure, and findings. The extracted data were tabulated using MS Excel 2010.

Data were extracted, including four domains:

1. Identification of the study (article type; journal type; author; country of the study; language; publication year; host institution of the study);
2. Methodological characteristics (study design; study objective; research question; sample characteristics, e.g., sample size, sex; age, statistical analysis);
3. Main findings;
4. Conclusions.

The authors were approached for any missing data in the studies. The year of publication was used as the year of data collection from the studies, which did not report the year of data collection.

### 2.7. Quality Assessment

- Risk of Bias

After data extraction, the reviewers independently assessed the possible risk of bias among eligible studies using a specific protocol developed to analyze cross-sectional studies, the Newcastle–Ottawa Scale, which was adapted to the outcomes evaluated in this review along with the Joanna Briggs Institute (JBI) tool for the assessment of prevalence studies. Reviewers scored the papers that adequately fulfilled each methodological criterion and provided a score with a maximum of 9 points in the JBI tool and 10 in the Newcastle–Ottawa Scale, according to the parameters considered. Parameters that did not apply to this revision also received scores and entered the computation of final points as mentioned in Table 3.

### 2.8. Statistical Analysis

- Assessment of Heterogeneity

Dichotomous data were expressed as risk ratios with 95% confidence intervals. Continuous measurement scales or ordinal data were expressed using the mean and standard deviation. Heterogeneity was evaluated using the I^2^ test with an alpha of 0.10. Review Manager (Revman V.5.3) was used for data synthesis. Meta-analyses were conducted by considering the prevalence of dental caries and periodontal disease as outcomes. Sensitivity analyses were also performed, with each study excluded sequentially. A Forest plot was used to verify the possible presence of publication bias.

## 3. Results

A systematic literature search using keywords and MESH search terms was conducted using PubMed (n = 113), Google Scholar (n = 1856), and EBSCO (n = 57) online databases. Of the 20 included studies in the current systematic review, 18 studies [8,10,11,12,13,14,15,16,17,18,19,20,22,23,24,25,26,27] were conducted in India while 1 study [9] was conducted in Malaysia and 1 study [21] was conducted in Iowa City.

**Figure 2:**
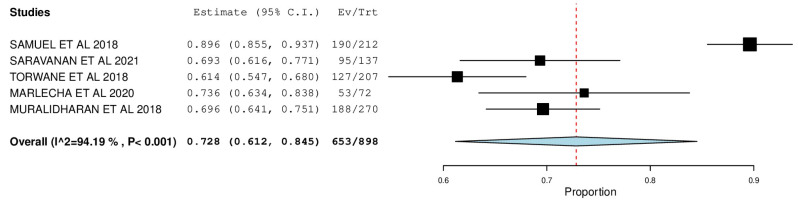
Forest plot depicting the prevalence of decayed teeth.

**Figure 3:**
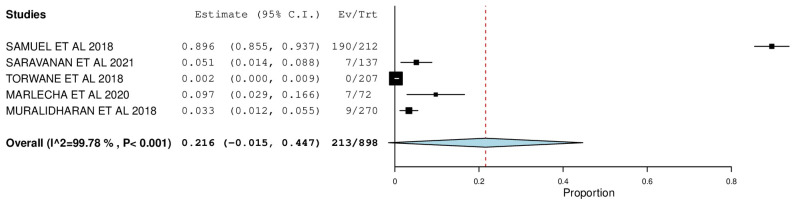
Forest plot depicting the prevalence of filled teeth.

**Figure 4:**
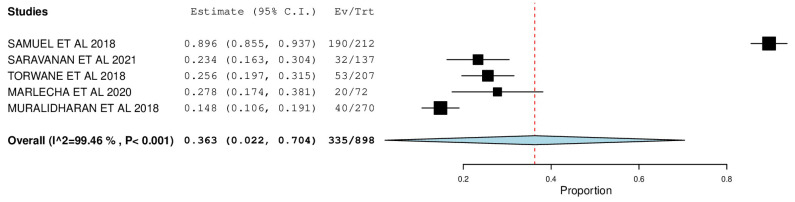
Forest plot depicting the prevalence of missing teeth.

**Figure 5:**
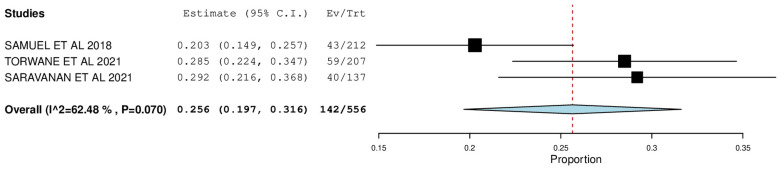
Forest plot depicting the prevalence of oral mucosal lesions.

**Figure 6:**
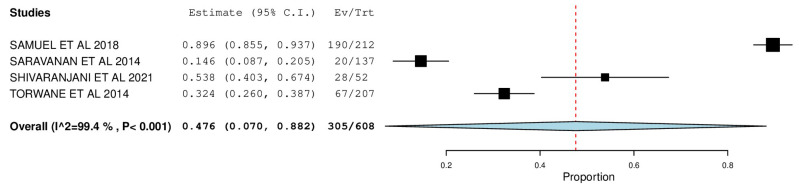
Forest plot depicting the prevalence of periodontal status as measured by pocket probing depth.

**Figure 7:**
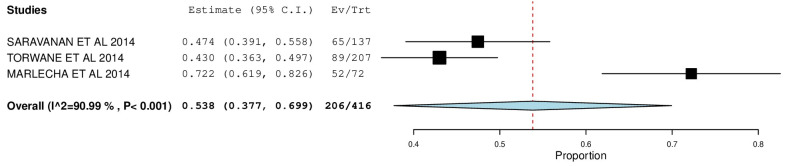
Forest plot depicting the prevalence of oral hygiene status as measured by the presence of calculus.

Figure 2, Figure 3, Figure 4, Figure 5, Figure 6 and Figure 7 depict the main results of the meta-analyses regarding the prevalence of dental caries, periodontal disease, oral mucosal lesions, and calculus, respectively.

The overall mean difference for decayed teeth was reported in 5 studies, which was 0.72, having a precision of 0.63 to 0.84. The results obtained were statistically significant (*p*-value < 0.05) (Figure 2).

The overall mean difference for filled teeth was reported in 5 studies, which was 0.36, having a precision of 0.02 to 0.70. The results obtained were statistically significant (*p*-value < 0.05) (Figure 3).

The overall mean difference for missing teeth was reported in 5 studies, which was 0.22, having a precision of −0.01 to 0.44. The results obtained were statistically significant (*p*-value < 0.05) (Figure 4).

The overall mean difference for oral mucosal lesions was reported in 3 studies, which was 0.25, having a precision of 0.19 to 0.31. The results obtained were not statistically significant (*p*-value > 0.05) (Figure 5).

The overall mean difference for periodontal status was reported in 4 studies, which was 0.47, having a precision of 0.07 to 0.89. The results obtained were statistically significant (*p*-value < 0.05) (Figure 6).

The overall mean difference for oral hygiene status was reported in 3 studies, which was 0.53, having a precision of 0.37 to 0.69. The results obtained were statistically significant (*p*-value < 0.05) (Figure 7).

Heterogeneity among the included studies was observed in both analyses (*p* < 0.001), and the analyses were considered to have high heterogeneity based on the I^2^ test (prevalence of decayed teeth—I^2^ = 94.19%; prevalence of missing teeth—I^2^ = 99.46%; prevalence of filled teeth—I^2^ = 99.78%; prevalence of periodontal disease—I^2^ = 99.4%; prevalence of oral mucosal lesions—I^2^ = 62.48%; prevalence of calculus—I^2^ = 90.99%.

### 3.1. Oral Mucosal Lesions

In the analysis of the included studies, the pooled prevalence of oral mucosal lesions was 25.6% (95% CI: 0.197, 0.316). The forest plot depicting the proportion of prevalence of the lesions in each study indicated that the study by Saravanan et al. had the highest prevalence—29.2% (95% CI: 0.216, 0.368), and that by Samuel et al. had the lowest—20.3% (95% CI: 0.149, 0.257). According to Samuel et al. [22], a more significant proportion of the population was affected with leukoplakia (26%), which was similar to findings put forth by Torwane et al. [25] (28.90%), with leukoplakia being the major lesion (7.7%). However, while Saravanan et al. [17] depicted an overall occurrence close to that reported by Torwane et al. (29.2%), his target population was significantly affected by candidiasis (13.9%).

### 3.2. Periodontal Status

Five studies were included in studying periodontal status via a forest plot with a pooled prevalence of 47.6% (95% CI: 0.070, 0.882). Samuel et al. reported the highest prevalence—89.6% (95% CI: 0.855, 0.937), and Saravanan et al. reported the lowest—14.6% (95% CI: 0.087, 0.205). The Calculus Index measured the presence of the calculus to estimate the target population’s oral hygiene status. A forest plot was designed with a pooled prevalence of 53.8% (95% CI: 0.377, 0.699). Torwane et al. reported the lowest prevalence—43% (95% CI: 0.363, 0.497), and Marlecha et al. reported the highest—72.2% (95% CI: 0.619, 0.826). Kalyan et al. [10] reported a significant prevalence of gingivitis (98.9%) and periodontitis (94.27%) among transgender persons, which was in congruence with findings by Torwane et al. [8] (92.8%) and Saravanan et al. [17] (83%).

### 3.3. Dental Caries

The percent DMF among the included studies was meta-analyzed to study the caries experience among the transgender population. In congruence with the forest plot, the highest prevalence was reported by Samuel et al.—D: 89.6% (95% CI: 0.855, 0.937), M: 89.6% (95% CI:0.855, 0.937), F: 89.6% (95% CI: 0.855, 0.937) and the lowest prevalence was reported by Torwane et al.—D: 61.4% (95% CI: 0.547, 0.680), M: 25.6% (95% CI: 0.197, 0.315), F: 00.2% (95% CI: 0.000, 0.009), with the pooled prevalence of D, M, and F being 72.8% (95% CI: 0.612, 0.845), 36.3% (95% CI: 0.022, 0.704), and 21.6% (95% CI: −0.015, 0.447), respectfully. A noteworthy conclusion one can draw from this forest plot is that while the treatment needs of this population seem high, the proportion of filled teeth is comparatively very low, which indicates poor utilization of dental services and inadequate knowledge regarding oral health diseases, leading to accumulated oral health problems that are majorly left untreated.

### 3.4. Risk of Bias Assessment

Of the 20 studies, 4 studies depicted 9/9 (100%) scores [10,18,22,27] having low risk of bias and strong methodological rigor. Nine studies [8,9,14,15,16,19,23,24,25] depicted 8/9 (88.8%) scores showing moderate risk of bias but were still reliable. Six studies had 7/9 (77.7%) scores [11,12,13,17,21,26] having moderate-to-high risk of bias, possibly due to missing methodological elements and only one study with 66.6% [20] showed high risk of bias, indicating concerns about study quality.

- Expected Heterogenous Variability in Global Meta-Analyses

The observed heterogeneity (I^2^ values above 90%) in this systematic review and meta-analysis reflects the inherent variations in study populations, methodologies, and reported outcomes across the included studies. However, rather than being an indication of methodological flaws, such heterogeneity is expected in a global synthesis of oral health data for transgender individuals, a population that faces diverse socio-cultural, economic, and healthcare-related barriers across different regions.

### 3.5. Diverse Study Designs and Populations

The included studies span multiple countries, each with different healthcare systems, cultural perceptions of transgender health, and accessibility to oral healthcare services. Such diversity naturally results in variability in reported oral health outcomes, contributing to heterogeneity. However, this is not necessarily a weakness but rather an expected feature of global epidemiological studies. Some studies used clinical examinations by trained professionals, while others relied on self-reported oral health status, introducing variability in outcome assessment.

### 3.6. Variability in Oral Health Determinants

The transgender population experiences unique challenges such as gender-affirming hormone therapy (GAHT), social determinants of health, and healthcare disparities, which differ significantly across studies. These factors influence oral health outcomes and contribute to heterogeneity.

### 3.7. Statistical Handling of Heterogeneity

While I^2^ values above 90% indicated substantial heterogeneity, random-effects models were employed to account for between-study variability, ensuring robust effect size estimates. Furthermore, sensitivity analyses were conducted to assess the impact of individual studies, ensuring that no single study disproportionately influenced the pooled results.

Sensitivity analysis was performed as part of our methodological rigor to assess the stability of the pooled effect sizes by removing high-variance studies. However, the results indicated no significant deviation from the primary meta-analysis outcomes, confirming that the overall findings were robust.

To ensure that the high heterogeneity did not compromise the validity of pooled estimates, the following statistical methodologies were rigorously applied:

Random-Effects Model (DerSimonian and Laird method) was deployed, which assumes that effect sizes vary across studies due to real differences rather than sampling error alone. Removing studies based on variance alone may introduce bias, as heterogeneity in effect sizes is expected due to real-world differences in transgender populations’ oral health across regions.

Leave-One-Out Sensitivity Analysis was performed to assess whether any single study disproportionately influenced the results. The effect sizes remained stable across iterations, confirming robustness. We systematically re-ran analyses by omitting each study individually to test its influence on the overall results. No single study was found to disproportionately impact the pooled estimates. This supports the robustness of our findings, negating the necessity of excluding high-variance studies arbitrarily.

Tau-Squared (τ^2^) Estimation and Prediction Intervals were calculated to quantify between-study variance beyond I^2^. This provides a more accurate representation of heterogeneity than I^2^ alone.

- Reporting Limitations and Data Constraints Regarding Subgroup Analysis

Lack of Stratified Data: Many of the included studies did not provide stratified results based on age, region, or access to oral healthcare, preventing meaningful subgroup analyses. Conducting subgroup analyses on limited sample sizes would introduce small-study bias and reduce statistical power, leading to unreliable conclusions.

Heterogeneous Reporting of Key Covariates: Some studies provided aggregate data for cisgender and transgender individuals combined, limiting our ability to extract transgender-specific subgroup data.

## 4. Discussion

In light of transgender persons being deliberated as a marginalized population facing extensive stigmatization and discrimination in the field of dental health care with increased frequency of unmet dental needs, the current systematic review maps the oral health and hygiene status of transgender persons.

The results of this systematic review strongly support the hypothesis that transgender individuals face significant oral health disparities due to systemic inequities. Across multiple studies, we observed patterns of poorer oral hygiene, higher rates of untreated dental diseases, and lower utilization of dental services among transgender populations. These findings align with broader public health research indicating that social determinants—such as discrimination, economic marginalization, and limited access to competent healthcare—play a crucial role in shaping health outcomes for transgender individuals.

In relevance to the 20 studies narrowed down to discern the oral health and hygiene status of transgender persons, four studies depicted a significant prevalence of oral mucosal lesions among them, which could be attributed to transgender persons of lower socioeconomic status and comparatively ill-educated being considerably prone to substance abuse in the form of tobacco and alcohol, as stated in the studies conducted by Stronks et al. [38] and Dangi et al. [39]. In a study performed to analyze the relationship between substance abuse and the occurrence of oral mucosal lesions, Mehrotra et al. [40] found that smokers and chewers among their target population were more afflicted than non-users. Samuel et al. stated that around 87.9% to 92.6% of his sample population were slum dwellers and relied on begging as their source of income, while 99% of the sample population of Torwane et al. belonged to a lower socioeconomic class.

Six studies reported that the dental caries experience ranged from 69.3%, as in that by Saravanan et al. [17], which was in congruence with those by Torwane et al. [8] (67.3%) and Muralidharan et al. [16] (69.6%) but was slightly higher in statistics in those by Marlecha et al. [20] (73.6%) and Ovia et al. (87%). Several factors could contribute to this population’s ubiquitous prevalence of dental caries. Increased frequency of sugar intake was standard in the studies by Torwane et al. and Saravanan et al., an essential parameter that should be considered while assessing dental caries. The population groups of these studies reported poor utilization of dental healthcare facilities, lower socioeconomic status, and inadequate knowledge regarding oral healthcare, including poor practices involving materials used to brush their teeth and the frequency of brushing. In accordance with the studies quoted above, a scarce population of only 26.9 to 42.3% preferred visiting a dentist, coupled with a lack of transgender-friendly dental facilities, discrimination in dental settings, and accumulated dental treatment needs due to unaffordability could condone the susceptibility of this population to dental decay. These factors were akin to the studies by Farah et al. [9], Prasanth et al. [13], Kumar et al. [14], and Shivaranjan et al. [18].

A noteworthy finding among all of the studies included in the systematic review was that the majority of them focused on the periodontal status of the transgender population. The presence of bleeding gums and calculus, markers of poor periodontal status, were reported by four studies, with occurrence ranging from 8.8 to 69% for bleeding gums and 43 to 72.2% for calculus. There was a broad difference in results computed for loss of attachment among transgender persons in studies by Saravanan et al. [17] (16.8%) and Marlecha et al. [20] (40.3%), which could be the fact that 60% of Marlecha et al.’s sample population had never visited a dentist before, leading to untreated and accumulated dental needs. Out of all the studies reporting pocket depth, Torwane et al. had the highest prevalence rate (32.4%) when compared to others (Shivaranjani et al.—15%, Saravana et al.—14.6%), which can be associated with greater tobacco use (90.3%) among its sample population. In consideration of the findings from the abovementioned studies, several factors influence a more significant proportion of transgender persons being affected by periodontal diseases, including increased stress-cortisol levels (Shivaranjani et al. [16]), deleterious habits such as tobacco chewing and smoking, which in turn lead to increased levels of periodontal pathogens in shallow and deep periodontal pockets, altered neutrophil chemotaxis, phagocytosis, and oxidative burst, increased TNF–α and PGE2 levels in gingival crevicular fluid (GCF), neutrophil collagenase and elastase in GCF, and production of PGE2 by monocytes [41]. Considering these findings, it is imperative to understand that oral health is linked to overall health and that poor dental health is associated with high levels of inflammation, poor diet quality, and conditions such as disability, diabetes, and increased risk of cardiovascular disease and pneumonia. Various factors like bacteremia, the release of inflammatory mediators, acute phase reactants, and alterations in immune responses could be possible links between the high prevalence of dental diseases and deteriorating overall health [42,43].

Inadequate knowledge regarding oral conditions, reluctance to visit a dentist, and poor oral hygiene further worsen periodontal status, as also reported by L. Ekanayake in a study conducted to assess the correlation between tobacco use and oral hygiene as a risk indicator for periodontitis [44].

The systematic review sought to standardize oral hygiene practices among transgender people since poor oral hygiene is the root cause of declining oral health. Nine out of the twenty included studies mentioned findings about the knowledge, attitude, and practice of transgender persons regarding the importance of oral hygiene. Farah et al. [9], Kumar et al. [14], Hongal et al. [15], and Prasant et al. [13] reported that their target populations were significantly aware of the importance of brushing, and 87.8% brushed their teeth at least once daily. Moreover, 65.3 to 86.1% of them preferred a toothbrush and toothpaste to clean their teeth (Kumar et al., Prasant et al., Saravanan et al., Kalyan et al., and Hongal et al.). Oral hygiene status was further measured with the help of the OHI-S index among four studies, of which Prasanth et al. reported that only 17% of transgender persons had good oral hygiene and 40% of them did not, which was similar to the findings by Kaur et al. in their study to assess the oral hygiene status of mentally and physically challenged individuals [45].

Comparing the oral health status of transgender and non-transgender populations reveals some significant differences. In a study conducted by Luiz et al. assessing the oral health related quality of life in the LGBTIQ+ population, 44.8% of transgender individuals reported frequent or severe oral health issues, whereas only 28.3% of cisgender individuals experienced similar issues. This difference was primarily attributed to transgender individuals with lower educational levels, lower family income, difficulty accessing dental treatment, and suicidal ideation or mental health issues as compared to the cisgender population [46].

Therefore, extensive research has yet to be conducted to decipher the oral health needs of the transgender community, how their oral health requirement varies, and how, as a health care provider, one can assist them adequately since one’s oral hygiene practices do influence one’s vulnerability to oral disease.

Of all of the included studies, only Marlecha et al. assessed malocclusion among transgender persons, with 80.55% of them having class 1 malocclusion and the rest having class 2 and class 3 malocclusion. Scarce data on such findings may be due to the fact that aesthetic considerations are seldom a priority among this community and that the collected data may be insignificant compared to other populations. Another unique study assessed the palatal rugae pattern among the transgender population. Palatoscopy is used in forensic sciences to determine a person’s identification, as it mirrors a person’s genetic makeup. The findings revealed that the rugae length was lesser and wavier, was more forwardly and backwardly directed, and had diverging rugae compared to the male and female control populations. This may serve as a step ahead in studying the genetic makeup and ethnic and racial differences of this community.

One of the inherent challenges of regular systematic reviews is the necessity of a fixed search period to ensure methodological rigor and reproducibility. In this study, our literature search was conducted until November 2021, meaning that any research published after this period was not included in our current analysis. Given the rapidly evolving nature of transgender health research, we acknowledge that more recent studies may offer additional insights that could further enrich our current findings. However, adhering to a pre-specified search window was critical to prevent selective reporting bias and maintain transparency in evidence synthesis. Since this is a living systematic review, newly published data in real time would be periodically updated and published (biennially or triennially, depending on the incidence of fresh publications), ensuring that emerging evidence continues to inform clinical practice and policy development.

Quite a few of the included studies did end up using the incorrect terminology and gender insensitive and no-inclusive discriminatory verbiage for the target population [17,22,25].

While we recognize the importance of distinguishing between different transgender subgroups (e.g., transgender women, transgender men, non-binary individuals, gender-diverse populations), many of the included studies did not stratify their data accordingly. This limitation prevented a statistically meaningful subgroup analysis without introducing significant bias due to small sample sizes within subgroups.

Given that oral health disparities among transgender individuals are largely influenced by systemic healthcare access issues rather than gender identity subcategories alone, aggregating the data into a single transgender category was deemed to be the most methodologically sound approach.

While attempts have been made in isolation to ascertain the oral health needs of the transgender population, most of them end up being thesis or research-oriented, offering minimal translational service for their treatment needs.

### 4.1. Oral Health Disparities Between Transgender and Cisgender Populations

Oral health disparities exist within all marginalized populations, but transgender individuals face unique structural, social, and medical challenges that exacerbate these disparities. To better understand these inequities, it is important to contrast transgender-specific factors with those affecting the broader cisgender population. This living systematic review helps bridge the knowledge gap between cisgender and transgender oral health disparities and lays the groundwork for future research that can quantitatively compare these populations in a matched-study format.

- *Prevalence and General Trends*

Studies on cisgender populations indicate that oral health disparities are largely driven by socioeconomic factors, healthcare access, education, and behavioral habits [46]. By contrast, transgender populations experience these same disparities in addition to gender identity-related stressors, such as discrimination in healthcare settings, stigma, and lack of culturally competent providers [35]. Several population-based studies indicate that cisgender individuals have greater access to preventive dental care, lower rates of untreated oral disease, and better oral health literacy than transgender individuals, particularly in countries where transgender-inclusive healthcare policies are lacking [3].

- *Social Determinants of Health (SDOH) and Their Role in Oral Health Disparities*

Healthcare Access: Cisgender individuals generally experience fewer barriers to healthcare compared to transgender individuals, who often report denial of treatment, lack of gender-affirming care, and fear of discrimination from dental professionals.

Economic Stability: Transgender populations are disproportionately affected by unemployment and economic instability, limiting their ability to afford dental services, whereas cisgender individuals have higher rates of employment-linked dental insurance coverage.

Psychosocial Stress and Behavioral Differences: The minority stress theory suggests that transgender individuals are at higher risk for engaging in adverse health behaviors (e.g., smoking, substance use) due to social marginalization, which contributes to increased periodontal disease and caries prevalence. These behavioral patterns differ significantly from those of cisgender individuals, who do not experience gender-related stressors at the same intensity.

- *Inclusion of Contextual Data from Existing Literature*

Although this living systematic review did not perform a direct comparative analysis between transgender and cisgender populations due to data limitations, we integrated existing evidence on cisgender oral health trends to better contextualize the findings.

Caries and periodontal disease prevalence in cisgender populations studies on global oral health indicate that dental caries and periodontal disease remain prevalent among cisgender individuals, particularly among lower-income groups. Population-based studies in high-income countries suggest that cisgender adults report lower rates of untreated caries than transgender individuals, likely due to greater access to routine dental care. By contrast, transgender individuals often experience higher levels of untreated oral diseases due to financial constraints and discrimination in dental settings.

#### 4.1.1. Comparison of Dental Visit Frequency and Preventive Care Utilization

Cisgender individuals are more likely to undergo regular dental checkups and receive preventive treatments, such as fluoride application and professional cleaning. Among transgender populations, studies indicate a significantly lower frequency of dental visits, often due to fear of being misgendered or receiving inadequate care from uninformed dental professionals. The inclusion of studies from lower-income countries suggests that oral health disparities among cisgender individuals are often linked to economic barriers, whereas transgender individuals face both economic barriers and additional gender-related healthcare discrimination.

There is a need for a targeted and focused comprehensive program ranging from care to cure to meet and explore the oral health needs of the transgender population. Translational and implementation steps should be adopted to carry forward this research data and fabricate the desired treatment needs that these sections of the population need. Incorporating culturally competent didactic and clinical learning experiences into the education of future oral health professionals may enhance the delivery of relevant and high-quality health care to the minority transgender population [47].

A recent study [48] contributed valuable insights into the oral health challenges faced by transgender populations. While this study was published around the same time as our search period, its findings align with the broader trends identified in our review, reinforcing the systemic disparities affecting this community. Although it was not included in our current meta-analysis due to predefined search parameters, its relevance highlights the importance of ongoing research in this domain. Since this is a living systematic review, further studies will be updated to this metaset periodically.

#### 4.1.2. Specific Recommendations to Improve the Field

Need for Comparative Studies—Future studies should be designed to include both transgender and cisgender populations in the same analysis while controlling for key confounding variables such as socioeconomic status, insurance coverage, access to healthcare services, and comorbid health conditions (e.g., HIV, diabetes, smoking status). Studies that fail to account for these confounders risk over- or underestimating the true disparities between transgender and cisgender populations.

Need for Matched-Control Studies—To obtain statistically valid and generalizable comparisons, future research should implement matched-control study designs, in which transgender and cisgender individuals are matched on relevant demographic and socioeconomic factors to ensure comparability. Stratified analyses by gender identity (transgender women, transgender men, non-binary individuals) should be conducted to explore subgroup-specific disparities. Large-scale population-based cohort studies are needed to assess long-term oral health trends among transgender individuals in comparison to their cisgender counterparts.

Call for Culturally Competent Oral Health Interventions—Public health programs, such as the Kartavya model by Kumar et al. [3], should incorporate targeted outreach and education to address the oral health disparities in transgender communities, ensuring equitable access to preventive and restorative dental care and proper salutations without pre-emptive misgendering or using culturally inappropriate terminologies. Dental health professionals should receive gender-affirming training to improve the oral healthcare experience for transgender individuals Dental professionals and policymakers must work collaboratively to create gender-affirming oral health initiatives that address the unique needs of transgender individuals.

One limitation of this systematic review is the variability in diagnostic criteria used across studies to define oral diseases such as periodontitis. Some studies relied on standardized guidelines (e.g., CDC/AAP case definitions), while others used broader or unspecified clinical assessments. Such discrepancies can lead to differences in reported prevalence and contribute to heterogeneity in our findings. To account for this, we conducted heterogeneity assessments (I2 statistics) and, where applicable, sensitivity analyses to evaluate the impact of diagnostic variations on pooled estimates. Despite these methodological differences, the overall trends in oral health disparities among transgender individuals remain consistent across studies, emphasizing the need for standardized diagnostic protocols in future research.

To improve the comparability of findings in future research, there is a pressing need for standardized diagnostic criteria for oral diseases, particularly in marginalized populations such as transgender individuals. Future studies should aim to adopt uniform case definitions (e.g., CDC/AAP for periodontitis) and ensure examiner calibration to minimize inter-study variability. Additionally, the implementation of globally recognized oral health assessment protocols, as outlined by the WHO, could enhance data consistency across different geographic regions and healthcare settings. Establishing these standards will allow for more precise cross-cultural comparisons and facilitate evidence-based policymaking to address oral health disparities.

## 5. Conclusions

This living systematic review and meta-analysis underscores the indigent oral health and hygiene status of the global transgender population, reinforcing the growing body of evidence on transgender health disparities. By adopting a dynamic and evolving approach, this review enables real-time tracking of trends, advancements in dental care, and the impact of shifting healthcare policies, ensuring that transgender oral health remains a continuously updated research priority.

Our findings validate the disproportionately poorer oral health outcomes experienced by transgender individuals, largely driven by systemic inequalities. These disparities highlight an urgent need for targeted research, policy reforms, and structural interventions to achieve equitable access to oral healthcare worldwide.

As authors, we believe that there is an urgent need to develop preventive and curative modules that aim at their inclusivity and acceptability in healthcare facilities, thereby appraising their oral health-related quality of life. This will not only enhance their overall health and tenacity but also instill in them positive dental health behavior, overarching the equitable ethos of establishing affirmative, accessible, acceptable, and affordable oral healthcare.

## Figures and Tables

**Table 1:** Characteristics and details of included studies.

S. No	Author/ Year	Country	Number of Participants (N)	Parameters Assessed	Outcomes (Expressed as Percentages and/or Mean ± Standard Deviation)
1	Arjun et al. [8]	Bhopal, Madhya Pradesh, India	N = 207	Visit to the dentist Dental facility availability Frequency of sugar intake DMFT ^1^ index Treatment needs	GENDER (%) Visit to the dentist—67 (32.4) Dental facility availability Government hospital—11 (5.3) Private clinic—173 (84) None—14 (6.8) Do not know—8 (3.9) Frequency of sugar intake Everyday—8 (3.9) Several times a week—30 (14.5) Once in a week—26 (12.6) Rarely—88 (42.5) Never—55 (26.6) D ^2^—127 (61.4) M ^3^—53 (25.6) F ^4^—0 DMFT total—139 (67.1) Treatment needs Fissure sealant—45 (21.7) One surface filling—122 (58.9) Two surface filling—40 (19.3) Pulp care and restoration—38 (18.4) Extraction—70 (33.8) Need for any other care—76 (36.7)
2	Farah et al. [9]	Terengganu state, Malaysia	N = 100	Habits Discrimination during dental treatment Frequency of brushing teeth	N (%) Smoking status 52 (52.5) Alcohol consumption 11 (11.1) Discrimination during dental treatment 21 (21.9) Frequency of brushing teeth Once daily—10 (10.3) Twice daily—52 (53.6) Thrice daily—30 (30.9) More than thrice daily—5 (5.2)
3	Kalyan et al. [10]	Central Gujarat	N = 384	Periodontal pocket depth Clinical attachment loss Oral hygiene Habits	Mean value of Periodontal pocket depth—3.12 ± 0.89 Clinical attachment loss—2.88 ± 0.74 N (%) Cleaning of teeth with Toothbrush—302 (78.64) Finger/stick—82 (21.35) Material used for cleaning Toothpaste—381 (99.2) Others—03 (0.78) Habit Smokeless tobacco—142 (36.97) Smoking tobacco—44 (11.45) Both smoking and smokeless tobacco—198 (51.56) Frequency of tobacco Once a day—16 (4.16) Multiple times in a day—368 (9.37)
4	Saravanan et al. [11]	Chennai city	N = 137	Oral Hygiene Habits Personal Habits Periodontal status	N (%) Oral Hygiene Habits a. Cleaning Toothbrush—86.1 Finger-11.7 Toothbrush & finger—11.8 Others—0.7 b. Materials Toothpaste—76.6 Tooth powder—16.8 Toothpaste & tooth powder—5.8 Others—0.8 Personal Habits Alcohol No—37.2, Yes—62.8 Gutka No—65.0, Yes—35.0 Tobacco No—70.1, Yes—29.9 Smoking No—93.4, Yes—6.6 Pan No—94.2, Yes—5.8 Periodontal status Healthy—27 Bleeding—8.8 Calculus—47.4 4–5 mm—12.4 6 mm—2.2 Excluded—2.2
5	Syed et al. [12]	Chennai city	N = 165	OHI ^5^ Calculus index Clinical attachment loss Pocket depth	Mean ± S.D OHI Index—2.69 ± 2.62 Calculus index—2 ± 2.35 Clinical attachment loss (CAL)—2.39 ± 0.490 Pocket depth (PD)—2.41 ± 0.4
6	Prasanth et al. [13]	Kancheepuram district	N = 75	OHI-S ^6^ Knowledge related to oral health	N (%) OHI—S Good—22.7 Fair—37.3 Poor—40 Cleaning of teeth Once in a week—14.7 Many times in a week—5.3 Once in a day—57.3 More than once a day—22.7 Material used to clean the teeth Toothpaste with brush—82.7 Tooth powder—13.3 Finger—4.0 Causes of dental caries Toothpaste without fluoride—56.0 Frequent use of sugar—29.3 Causes for bleeding during brushing Inadequate bruising—8.0 Don’t know—6.7 Natural physiological phenomenon—14.7 Periodontal disease—13.3 Brushing too hard—13.3 Systemic diseases—18.7 Don’t know—40.0 Measures that prevent oral diseases Application of fluoride—9.3 Tooth scaling—44.0 Don’t know—46.7 Systemic diseases that may be related to oral diseases Diabetic—13.3 Hypertension—12.0 Cancer—20.0 Other diseases—17.3 Don’t know—37.3
7	Kumar et al. [14]	Bhubaneswar	N = 205	Oral hygiene habits	N (%)- Rural/Urban Material used for cleaning the teeth Finger—14.3%/4.7% Neem—22.4%/8.4% Twig—27.6%/22.4% Tooth powder—13.3%/17.8% Toothpaste—13.3%/43% Brushing of teeth regularly Yes—87.8%/91.6% No—12.2%/9.4% Visit to the dentist Once—12.2%/25.2% Twice—3.1%/17.8% Never—81.6%/43.9% Other—3.1%/13.1%
8	Hongal et al. [15]	Bhopal City, Madhya Pradesh	N = 207	Knowledge, Attitude, Practice regarding oral hygiene	N (%) KNOWLEDGE Good oral health can improve general health- Yes 168 (81.2), No 19 (9.2), Don’t know 20 (9.7) Main cause of tooth decay Sweet/wafers/biscuits/cakes—157 (75.8) Fresh fruit—7 (3.4) Raw vegetables—14 (6.8) Don’t know—29 (14) Tooth decay Tiny black spot in tooth—44 (21.3) Large hole in tooth—71 (34.3) Occurrence of pain—72 (34.8) Don’t know—20 (9.7) Cause of mouth cancer Usage of betel nut and betel quid—50 (24.2) Usage of tobacco—88 (42.5) Alcohol—21 (10.1) Don’t know—48 (23.2) ATTITUDE Condition of mouth Excellent—5 (2.4) Good—82 (39.6) Fair—38 (18.4) Poor—75 (36.2) Don’t know—7 (3.4) Ever visited a dentist for any problem Yes—67 (32.4) No—140 (67.6) Visit to dentist in last year Once—36 (17.4) Twice—4 (1.9) Three times—0 More than three times—1 (0.5) Not visited—166 (80.2) Would like to treat the deep painful decay by Root canal treatment—48 (23.2) Removal of teeth—107 (51.7) At present postpone the treatment—4 (1.9) Don’t know—48 (23.2) PRACTICES Cleaning of teeth with Toothbrush—176 (85) Finger—31 (15) Chew stick/neem stick—0 Others—0 Material use for cleaning of teeth Toothpaste/powder—205 (99) Charcoal—2 (1) Lime salt—0 Others—0 Frequency of brushing Once—181 (87.4) Twice—26 (12.6) After meal—0 Don’t know—0 Frequency of changing toothbrush 1–3 months—119 (57.5) 4–6 months—1 (0.5) 1 year and above—0 After wear—58 (20) Don’t know—29 (14) Use of any other oral hygiene aid Dental floss—4 (1.9) Inter-dental brush—0 Toothpick—27 (13) Mouth wash—1 (0.5) None—176 (86.3) Frequency of sugar intake Everyday—8 (3.9) Several times a week—30 (14.5) Once in a week—26 (12.6) Rarely—88 (42.5) Never—55 (26.6) Use of tobacco Smokeless tobacco—113 (54.6) Smoking tobacco—0 Both smokeless and smoking tobacco—74 (35.7) Total tobacco usage—187 (90.3) Frequency of tobacco usage Once in a day—6 (2.9) Many times a day—181 (87.4) Several times a week—0
9	Muralidharan et al. [16]	Pune	N = 270	DMFT ^7^	MEAN ± SD DT ^8^ —4.6741 ± 4.39724 MT ^9^ —0.2667 ± 0.96558 FT ^10^ —0.1407 ± 1.32835 DMFT—5.0778 ± 4.81377 N (%) Type of treatment required Pit and fissure sealant—0 One surface filling—159 (58.9) Two surface filling—46 (17.0) Pulp care and restoration—71 (26.3) Extraction—56 (20.7) Prosthetic need—66 (24.2)
10	Saravanan et al. [17]	Chennai city	137 participants	DMFT Sweet consumption Oral hygiene practices Dental visit Habits Oral mucosal lesions Periodontal status	Decayed teeth—69.3% Filled teeth—5.1% Missing teeth—23.4% Sweet consumption—83.2% Cleaning habits Toothbrush—86.1% Finger—11.7% Material used Toothpaste—76.6% Tooth powder—16.8% Dental visit—42.3% Personal habits Alcohol—62.8% Gutka—35% Tobacco—29.9% Smoking—6.6% Pan chewing—5.8% Oral mucosal lesions Candidiasis—13.9% Ulceration—12.4% Leukoplakia—1.5% OSMF—0.7% Lichen planus—0.7% Periodontal status Bleeding—8.8% Calculus—47.4% Pocket depth—14.6% Loss of attachment— 0–3 mm—83.2% 4–5 mm—12.4% 6–8 mm—2.2%
11	Shivaranjani et al. [18]	Puducherry	N = 52	Knowledge, Attitude, Practice regarding oral health Periodontal pocket depth Clinical attachment loss Bleeding Marginal gingival index	N (%) Oral health knowledge—PRE/POST 1 Good oral health leads to good general health—85.2/100 2 Sweets as the main cause of dental caries—61.1/92.6 3 Tiny black spot in the tooth indicates dental caries—35.2/89 4 Removal of teeth as the treatment of painful decay—31.5/83.3 5 Vigorous brushing can cause tooth sensitivity—46.3/88.5 6 Tobacco usage causes mouth cancer—46.3/61.1 7 Dental facilities in their locality—5.6/100 Oral health attitude 8 Unaware of bad breath—25.9/11.1 9 Visited dentist—28.8/90.7 10 No dentist visit last year—61.1/0 Oral health practices 11 Increased frequency of sugar intake—57.9/51.9 12 Using toothbrush—81.5/90.7 13 Using toothpaste—91.4/94.4 14 Habit of brushing twice a day—18.5/55.6 15 Change toothbrush every 3 months—33.3/74.1 16 Use of oral hygiene aids—5.6/33.3 Pre/Post MGI ^11^ —1.72 ± 0.80 0.93 ± 0.9 Bleeding site—21.80 14.70 PPD ^12^ 0—13/21 1—10/4 2—5/3 CAL ^13^ 0—31.1/56.78 1—32.1/24.7 2—24.3/5.2 3—0.31/1.23 4—7.4/5.56 X—0.63/2.48 9—4.03/3.1
12	Ovia et al. [19]	Chennai city	100	Oral hygiene	78%—brush teeth everyday 57%—use toothpick regularly 87%—have dental caries 69%—bleeding gums
13	Marlecha et al. [20]	Chennai city	N = 72	DMF ^14^, ROOT STUMPS, IMPACTED TEETH, ABRASION, ATTRITION, PLAQUE, CALCULUS, Loss of attachment, GINGIVAL INFLAMMATION, MALOCCLUSION	N (%) Without dental caries—26.4 With dental caries—73.6 Teeth missing due to dental caries—27.8 Filled teeth—9.7 Root stumps—29.2 Impacted teeth—15.3 Abrasion—44.4 Attrition—37.5 Presence of plaque—68.1 Presence of calculus—72.2 With gingival inflammation—38.9 With loss of attachment—40.3 Malocclusion findings Class 1—80.55 Class 2—12.50 Class 3—6.95
14	Olayinka et al. [21]	IOWA city	N = 769		The rate of oral HPV ^15^ was higher in gay and lesbian individuals (11.3%) relative to bisexual (8.6%) and heterosexual individuals (7.1%). There was a significant difference in self-reported oral health measures: bisexual and homosexual individuals had higher rates (40.9% and 35.8%, respectively) of self-reported fair/poor oral health compared to 27% in heterosexual individuals. Bisexual individuals were more likely to confront barriers to accessing dental care (30%) versus heterosexual adults (19%). Gay men reported a higher rate of a history of “bone loss around teeth” (35%) when compared to their heterosexual counterparts (11%).
15	Samuel et al. [22]	South India	N = 212	Periodontal Health: Pocket Depth Caries Experience: DMF ^16^ Lesions Tobacco use Previous dental visit Perceived Barriers HIV ^17^ Status	N (%) Periodontal Health Pocket Depth—190 Caries Experience Decayed Teeth—190 Missing teeth—190 Filled teeth—190 Lesions Candida—33 (17.3) Leukoplakia—5 (26) Lichen Planus—1 (0.5) Tobacco Pouch Keratosis—4 (2.1) Tobacco use—Yes—93.2, No—6.8 Form of Tobacco use Chewable—82.4 Smoked—0 Both—17.6 Previous dental visit—No—95.3, Yes—4.7 Perceived Barriers Non-Admittance—60.5 Economic—35.8 Others—3.7 Perceived OH Very poor—14.2 Poor—52.1 Neither—23.7 Good—8.4 Very good—1.6 N HIV Status—Yes—4, No—5, Don’t know—181
16	Sivaranjani et al. [23]	Puducherry and Cuddalore	N = 75	Periodontal pocket depth Clinical attachment loss	The mean PPD ^18^ and CAL ^19^ of participants were 4.06 ± 0.70 and 3.97 ± 0.68, respectively. The mean cortisol level was 6.02 ng/mL. A strong, positive correlation was observed between mean cortisol level and periodontal parameters assessed (probing depth and cortisol –r = 0.592, *p* = 0.000; clinical attachment loss and cortisol levels –r = 0.618, *p* = 0.000)
17	Saxena et al. [24]	Bhopal City, Madhya Pradesh	N = 48	Palatal rugae pattern	mean ± SD Number of rugae—11.1 ± 2.1 Rugae length Primary—7.14 ± 1.44 Secondary—3.79 ± 2.37 Fragmentary—0.31 ± 0.71 Rugae shape Straight—0.35 ± 0.56 Curve—3.60 ± 2.05 Wavy—6.89 ± 1.47 Circular—0.00 ± 0.00 Rugae direction Forwardly directed—6.25 ± 1.80 Backwardly directed—3.68 ± 2.27 Perpendicular directed—0.66 ± 0.85 Unification of rugae Converging rugae—0.29 ± 0.61 Diverging rugae—0.37 ± 0.68
18	Torwane et al. [25]	Bhopal City, Madhya Pradesh	N = 207	Oral mucosal conditions	N (%) Oral Mucosal Condition No condition—148 Malignant tumor—1 (0.5) Leukoplakia—16 (7.7) Lichen planus—2 (1.0) Traumatic ulceration—12 (5.8) Abscess—6 (2.9) Other conditions OSMF ^20^—20 (10) Smoker’s Palate—0 Pouch Keratosis—2 (1) Burn—0 Total—59 (28.5) Total population—207
19	Prasanth et al. [26]	Chennai city	N = 120	Assessment of Micronuclei in the exfoliated Buccal Mucosal Cells	The mean age of the population was 29 ± 4.60 years. While comparing the mean micronuclei count, it was significantly less (mean 5.37 with SD 1.12) among those have the habit of only chewing tobacco or pan. But alcoholic and alcohol with tobacco cases had higher counts (mean 9.27 with SD 4.12 and 7.10 with SD 4.32, respectfully).
20	Torwane et al. [27]	Bhopal City, Madhya Pradesh	N = 207	Periodontal Status Loss of Attachment	Periodontal Status Healthy—6.3% Bleeding—17.4% Calculus—43% Shallow pocket—22.7% Deep pocket—9.7% Loss of Attachment 0–3 mm—61.8% 4–5 mm—15.9% 6–8 mm—10.1% 9–11 mm—4.8% >12 mm—6.3%

^1^ Decayed, Missing and Filled Teeth, ^2^ Decayed, ^3^ Missing, ^4^ Filled, ^5^ Oral Hygiene Index, ^6^ Oral Hygiene Index Simplified, ^7^ Decayed, Missing, Filled Teeth, ^8^ Decayed teeth, ^9^ Missing teeth, ^10^ Filled teeth, ^11^ Marginal Gingival Index, ^12^ Pocket probing depth, ^13^ Clinical attachment loss, ^14^ Decayed, Missing and Filled, ^15^ Human papilloma virus, ^16^ Decayed, Missing and Filled, ^17^ Human immunodeficiency virus, ^18^ Pocket probing depth, ^19^ Clinical attachment loss, ^20^ Oral submucous fibrosis.

**Table 2:** List of excluded studies.

S. No.	Study	Reason for Exclusion
1	Tomasz Zyla et al. [28]	Literature Review
2	Roberts et al. [29]	Literature Review
3	Gheit et al. [30]	Not related to primary outcome
4	Michael R Kauth et al. [31]	Not related to primary outcome
5	Khalaf F Al-Shammari et al. [32]	Irrelevant study population
6	Segal et al. [33]	Review article
7	Macdonald [34]	Qualitative study
8	Russell et al. [35]	Literature Review
9	Parish et al. [36]	Qualitative study
10	Coulter et al. [37]	Literature Review

**Table 3:** Risk of bias assessment for included studies.

S. No.	Author (YR)	JOANNA BRIGGS Institute (JBI) Tool for Risk of Bias Assessment for Prevalent Studies											New Castle–Ottawa Quality Assessment Scale		
		1	2	3	4	5	6	7	8	9	Total	%	Selection	Comparability	Outcome
1	Arjun et al. (2018) [8]	1	1	-	1	1	1	1	1	1	8	88.8	***	*	**
2	Farah et al. (2020) [9]	1	1	-	1	1	1	1	1	1	8	88.8	***	NA	**
3	Kalyan et al. (2021) [10]	1	1	1	1	1	1	1	1	1	9	100	***	NA	**
4	Saravanan et al. (2014) [11]	1	0	-	1	1	1	1	1	1	7	77.7	**	*	**
5	Syed et al. (2017) [12]	1	0	-	1	1	1	1	1	1	7	77.7	**	*	**
6	Prasanth et al. (2020) [13]	1	1	-	0	1	1	1	1	1	7	77.7	**	NA	**
7	Kumar et al. (2021) [14]	1	1	-	1	1	1	1	1	1	8	88.8	***	*	**
8	Hongal et al. [15]	1	1	-	1	1	1	1	1	1	8	88.8	***	*	**
9	Muralidharan et al. [16]	1	1	-	1	1	1	1	1	1	8	88.8	***	NA	**
10	Saravanan et al. [17]	1	0	-	1	1	1	1	1	1	7	77.7	**	*	**
11	Shivaranjan et al. [18]	1	1	1	1	1	1	1	1	1	9	100	***	NA	**
12	Ovia et al. [19]	1	1	-	1	1	1	1	1	1	8	88.8	***	NA	**
13	Marlecha et al. [20]	1	-	-	0	1	1	1	1	1	6	66.6	**	NA	**
14	Olayinka et al. [21]	1	-	-	1	1	1	1	1	1	7	77.7	**	NA	**
15	Samuel et al. [22]	1	1	1	1	1	1	1	1	1	9	100	***	NA	**
16	Sivaranjani et al. [23]	1	1	-	1	1	1	1	1	1	8	88.8	***	*	**
17	Saxena et al. [24]	1	0	1	1	1	1	1	1	1	8	88.8	***	*	**
18	Torwane et al. [25]	1	1	-	1	1	1	1	1	1	8	88.8	***	*	**
19	Prasanth et al. [26]	1	1	-	0	1	1	1	1	1	7	77.7	**	*	**
20	Torwane et al. [27]	1	1	1	1	1	1	1	1	1	9	100	***	*	**

*, ** and *** Represent points awarded for meeting quality criteria.

## Data Availability

The data presented in this study are available upon request from the corresponding author.
